# All-optical switch exploiting Fano resonance and subwavelength light confinement

**DOI:** 10.1515/nanoph-2024-0644

**Published:** 2025-02-13

**Authors:** Quentin Saudan, Dagmawi A. Bekele, Meng Xiong, Kresten Yvind, Michael Galili, Jesper Mørk

**Affiliations:** DTU Electro, Technical University of Denmark, DK-2800 Kongens Lyngby, Denmark; Phanofi ApS, DK-2800 Kongens Lyngby, Denmark; NanoPhoton – Center for Nanophotonics, Technical University of Denmark, Kongens Lyngby, Denmark

**Keywords:** nanophotonics, nanocavity, Fano resonance, optical switching, extreme light confinement

## Abstract

We propose and experimentally demonstrate a small-mode volume bowtie cavity design for all-optical switching applications using a waveguide-cavity structure that exploits asymmetric Fano resonance lineshapes. The bowtie cavity has a mode volume that is five times smaller than conventional (H0-type) photonic crystal point-defect cavities enabling higher nonlinearity and faster switching. Blue and red-detuned Fano resonant devices based on bowtie cavity designs have been fabricated and characterized. Measured linear transmission spectra have been compared to coupled-mode theory models to extract key parameters such as Q-factors. Furthermore, all-optical switching at 2.5 Gbps have been demonstrated in a wavelength-conversion experiment.

## 1 Introduction

Optical cavities that strongly enhance light–matter interaction have enabled realization of several all-optical signal processing devices and applications including optical memory [1], optical switches [2], [3], [4], [5], optical diodes [6], [7], narrow-band filters [8], and semicondutor nanolasers [9], [10], [11], [12]. Point-defect cavities using photonic bandgap effects to confine light enable the realization of cavities with high quality factor and, simultaneously, small optical mode volume [13], [14]. Conventional point-defect cavities have mode volumes on the order of ≃2*V*_*λ*_ or larger, where *V*_*λ*_ ≡ (*λ*/2*n*)^3^, with *n* the refractive index of the material, and *λ* the wavelength of light. Here, *V*_*λ*_ is often denoted the diffraction-limited mode volume for dielectric cavities [15], although several designs [16], [17], [18] and recent experimental results [19], [20], [21] show that mode volumes much smaller than *V*_*λ*_ can be achieved. For optical switching, a smaller mode volume implies a stronger spatial concentration of light, enabling more efficient and faster switching. In particular, indium phosphide (In*P*) based photonic crystal membranes constitute an important platform for realizing such nanocavities and integrating them with active material and passive waveguides [22]. Additionally, In*P* is suitable for generating free-carriers using two-photon absorption with photon energies in the telecommunication wavelength range.

Optical signal processing functionalities have been improved by tailoring the lineshape of the optical resonance, so that the transmission through the structure is more sensitive towards shifts of the resonance [23]. The resonance is shifted due to the refractive index change induced by the optical pump (control) signal and depends on the particular physical mechanism at play in the material used. However, as pointed out in Ref. [24], the material used has less influence on the required power for switching than the resonant or travelling wave scheme used to enhance the interaction time between light and matter. Two of the commonly used characteristic lineshapes are the Lorentzian and the Fano lineshapes. In particular, the use of asymmetric Fano resonances have enabled improved optical switching characteristics [25]. Fano resonances are a manifestation of wave interference and arise in many different physical systems where a continuum of modes interacts with a discrete resonance [26]. Photonics offers a very rich platform, where Fano resonances has been explored in many different materials and configurations [27], [28], [29], [30].

**Figure 1:**
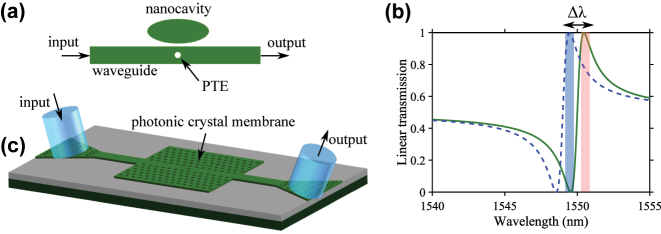
Device and transmission characteristic. (a) Schematic diagram of a Fano structure consisting of a nanocavity side-coupled to an optical waveguide. A partially transmitting element (PTE) is placed in the middle of the waveguide. (b) Illustration of all-optical switching using an asymmetric Fano resonance. The transmission spectrum from input to output, calculated using temporal-coupled-mode theory, are shown for the unshifted resonance (solid line) and the shifted resonance (dashed line). The spectral locations of the probe and pump signals are indicated by the blue and red shaded regions, respectively. (c) Schematic diagram of an integrated photonic crystal Fano device. Light is coupled in and out of the device using grating-assisted fiber-to-chip couplers. The waveguide and nanocavity shown in (a) are implemented in the photonic crystal membrane using a line-defect waveguide and a point-defect cavity.

Figure 1(a) shows a schematic diagram of the Fano structure we are considering. It consists of a nanocavity that is side-coupled to an optical waveguide. A partially transmitting element (PTE) placed in the middle of the waveguide determines whether the lineshape of the transmission spectrum from input to output is symmetric or asymmetric [31]. An asymmetric lineshape indicates the presence of paths of constructive and destructive interference between the discrete state and the continuum of states, depending on the wavelength, cf. Figure 1(b). All-optical switching devices rely on optically shifting the resonance using a control (or pump) optical pulse, which in turn changes the transmission of an optical signal (or probe) pulse. The pump signal generates free-carriers inside the nanocavity through two-photon absorption. The build-up of carriers inside the nanocavity changes the refractive index via the plasma effect, which in turn shifts the resonance wavelength of the nanocavity, thereby changing the transmission of the probe signal [32]. Figure 1(b) illustrates such a resonance shift (Δ*λ*), in which the spectral locations of the pump and the probe signals are indicated by red-shaded and blue-shaded regions, respectively. A comprehensive review on the use of photonic crystal Fano resonances for in-plane optical signal processing applications may be found in Ref. [25].

Several challenges have to be overcome before these all-optical switches can be applied in communication systems. One of these is the cross-talk between the switching and the switched signals, i.e., the pump and probe signals. As the probe and pump signals are in close spectral proximity, the probe signal suffers from in-band cross-talk. This issue can be addressed using multi-mode cavities [33], or photonic molecules [34], [35]. Another issue relates to the requirement that the pump-induced transmission change must relax back to the initial state before the next pump pulse arrives in order to avoid patterning effects [36]. The maximum operation speed of all-optical switches relying on index changes induced by free carriers is thus limited by the rate of carrier recombination. Several approaches have been proposed to increase the switching speed, such as employing a pn-junction in the nanocavity to sweep-out the free-carriers by applying a static electric field [37], and using doped semiconductors to increase the non-radiative recombination rate [4].

Recently, a new class of topolology-optimized nanocavities, denoted extreme dielectric confinement (EDC) cavities, which allow photon confinement well below the diffraction limit in dielectrics has been experimentally demonstrated [21], [38]. The optimal structures have a bowtie structure at the center, which act to concentrate the field due to the boundary conditions of Maxwell’s equations [39]. It was shown theoretically that such nanocavities may speed up the recovery dynamics significantly by relying on fast diffusion of the strongly localized carrier distribution [40]. Other designs employing super-cell tailoring also allow for strong spatial localization of the optical intensity but at multiple hot-spots [41], which is not suitable for switching.

In this paper, we present the design of a bowtie cavity incorporated inside a standard photonic crystal point-defect cavity, which is side-coupled to a line-defect photonic crystal waveguide forming a Fano resonance structure. This design allows simultaneously exploiting the strong light concentration in the bowtie cavity and the Fano resonance to improve the optical switching characteristics. The strong light concentration in the bowtie cavity thus increases the effective nonlinear index change induced by the pump signal and the asymmetric Fano spectrum increases the transmission change of the probe due to the nonlinear index change. The paper reports the first proof-of-principle investigations of such structures, including design optimization, experimental fabrication, linear device characterization, and a system experiment demonstrating wavelength conversion.

## 2 Bowtie cavity design

**Figure 2:**
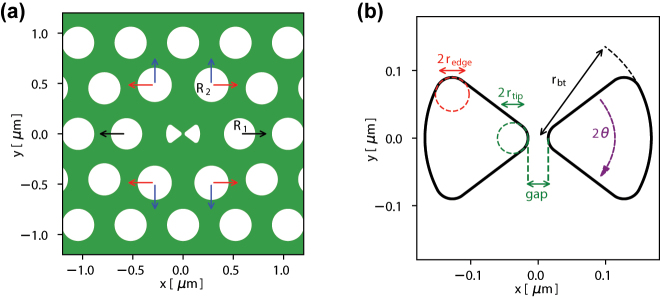
Design of bowtie cavity based on the photonic crystal membrane platform. (a) Point-defect H1 cavity with a bowtie at its center. The nearby holes are shifted to optimize the cavity mode, as indicated by *C*1 (black arrows), *C*2*x* (red arrows) and *C*2*y* (blue arrows), and their radii are scaled up or down to *R*1 and *R*2. (b) The central bowtie structure with identification of key parameters, such as bowtie tip radius, total radius of the bowtie, and the angle of the bowtie.

A design for a bowtie photonic crystal (PhC) nanocavity of H1 type with an embedded bowtie structure at its center is shown in Figure 2(a). Such a quasi-H1 type cavity is formed by replacing the center airhole with a bowtie structure. The design goal is to focus the H1 dipole mode into a mode with high electric field at the center of the bowtie. This cavity is designed for small-mode volume and high-quality factor using a two-step particle swarm optimization [42] relying on 3D finite difference time domain (FDTD) simulations. First, the bowtie structure is placed at the center of an H1 cavity, i.e., with one missing airhole, and the neighboring airholes were not scaled or shifted. The free parameters for the particle swarm optimization are the bowtie tip radius, the total radius of the bowtie, and the angle of the bowtie. We set the minimum values for the gap and the radius of curvature of the bowtie tip to be 30 nm and 20 nm, respectively. Although smaller tip radius would yield a smaller mode volume, it would not be feasible in our fabrication process based on electron beam lithography. A thorough explanation of the process flow is available in Ref. [43]. A small tip radius would also lead to a confinement of the major part of the field at the surface of the tip, which is undesirable due to scattering [19]. The figure of merit for this first optimization is the mode volume of the cavity evaluated at the center of the bowtie, and the spectral location of the resonance wavelength. After the first optimization, the following parameters are chosen: bowtie radius of curvature of *r*_edge_ = 25 nm, the gap between the bowtie 30 nm, the radius of curvature of the tip *r*_tip_ = 22 nm, the angle of the bowtie *θ* = 37°, and the total radius of the bowtie *r*_bt_ = 168 nm, cf. Figure 2(b).

The second optimization is performed on the H1 cavity with fixed bowtie structure designed in step one. In this case, the free-parameters for the particle swarm optimization are *C*1, *C*2*x*, *C*2*y*, *R*1 and *R*2 as shown in Figure 2(a). The lattice parameter *a* and the global airhole radius *r* of the photonic crystal are also considered as free parameters. The figure-of-merit to be maximized is the ratio of the intrinsic quality factor to the effective mode volume, *Q*_in_/*V*_eff_, subject to the condition that the resonance wavelength is located in the telecom C-band wavelength range. The final optimized parameters are a PhC lattice constant of *a* = 522 nm, radius *r* = 159 nm, *C*1 = 0.077*a*, *C*2*x* = 0.041*a*, *C*2*y* = 0.065*a*, *R*1 = 155 nm, and *R*2 = 170 nm.

**Figure 3:**
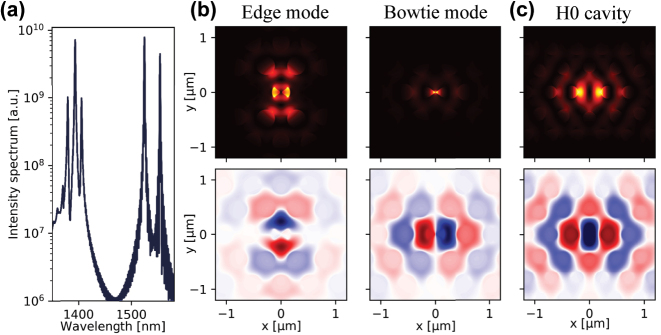
Simulated spectrum and mode profiles. (a) Optical spectrum of the field in the cavity obtained by 3D FDTD simulations. The electric field intensity |**E**|^2^ (top panels), and the real part of the magnetic field *H*_*z*_ (bottom panels) distributions for: (b) edge mode at 1,524.1 nm and bowtie mode at 1,553.4 nm of the bowtie cavity based on H1 cavity, and (c) the fundamental mode of an H0 cavity for comparison purpose.

Figure 3(a) shows the spectrum of the cavity field, obtained by 3D finite-difference-time-domain (FDTD) simulations, from which a number of modes can be identified. The modes around 1,550 nm have magnetic field profiles that match the two dipole modes of the H1 cavity, as illustrated in the bottom plots of Figure 3(b), which show the magnetic field-component *H*_*z*_, i.e., in the direction orthogonal to the membrane plane. Looking at the electric field profiles, we see that the dipole mode at 1,553.4 nm has a strongly enhanced electric field at the center of the cavity due to the bowtie structure, whereas the dipole mode at 1,524.1 nm is located at the edges of the bowtie. Hence, we refer to these modes as the bowtie mode and the edge mode, respectively. For comparison, we have plotted the field distributions of the fundamental mode of an H0 type cavity in Figure 3(c). Mentioned in numerous studies on photonic crystal cavities [1], [7], [32], the H0 cavity is the main comparative design helping us to benchmark our newly designed bowtie cavity.

The bowtie mode has a quality factor of *Q*_in_ = 5 × 10^4^, an effective mode volume of *V*_eff_ = 0.057(*λ*/*n*)^3^ (evaluated at the center of the bowtie cavity), and a nonlinear mode volume [44] of *V*_TPA_ = 0.723(*λ*/*n*)^3^. Compared to the fundamental mode of an H0 cavity, the bowtie cavity embedded in an H1 cavity has a mode volume that is five times smaller. Although, the intrinsic quality factor of the bowtie mode is four times smaller than the H0 cavity, it will not drastically impact the performance of the Fano resonance device as the total Q-factor, *Q*_tot_, will be dominated by the waveguide that it is coupled to. Simulations based on temporal coupled-mode theory (TCMT) model, discussed in ref. [26], [31], [45], suggest that a drop of the *Q*_in_ from 2 × 10^5^ to 5 × 10^4^, for a fixed waveguide Q-factor *Q*_wg_ = 1,600, would only result in a reduction of the *Q*_tot_ from 1,587 to 1,550, and a decrease of the extinction ratio (defined as the ratio of transmission maximum to transmission minimum of the Fano resonance) from 55 dB to 42 dB. Therefore, the impact of a low *Q*_in_ value is not expected to be significant for the overall performance of the optical switch.

**Figure 4:**
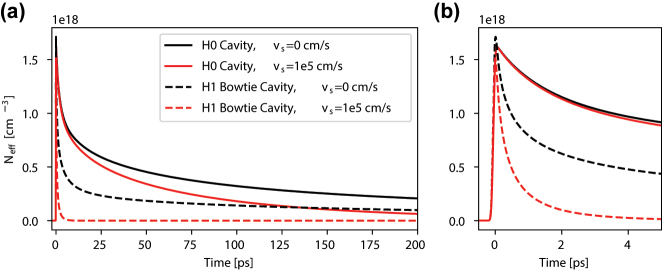
Carrier density dynamics. (a) The evolution of the effective electron carrier density *N*_eff_ for the fundamental mode of an H0 cavity (solid lines) and for the bowtie mode of the H1 bowtie cavity (dashed lines) for different surface velocity recombination *v*_*s*_. (b) Zoom-in of the dynamics during the first 5 ps.

Using the electric field profile of the optimized bowtie design, we calculated the spatial profile of free carriers generated by two-photon absorption. Subsequently, the dynamics of the carrier distribution due to drift and diffusion was simulated using the finite element method from the COMSOL semiconductor module and the results were compared to a reference H0 cavity design. We considered an In*P* membrane with a slight *n*-type background doping of 10^15^ cm^−3^. The carrier generation rate is adjusted so that the peak carrier concentrations *N*_eff_ of the two cavities are the same. As expected from the reduction of the mode volume, a shortening of the relaxation tail for the bowtie mode compared to the H0 cavity mode is obtained. This is seen in Figure 4 both in the absence of surface recombination and for a recombination velocity of 10^5^ cm/s. We notice that for the H0 cavity, the initial fast relaxation dynamics is unaffected by the surface recombination velocity, which is not the case for the bowtie mode. As both cavities have the same vertical dimension and surface recombination at the membrane interface, the difference must come from the surface recombination due to the photonic crystal airholes. Hence, we attribute it to the larger exposure of the bowtie mode to air–dielectric interfaces as compared to the H0 cavity mode. On the one hand, this greatly shortens the relaxation tail of the bowtie mode, reducing patterning effects and allowing for a higher modulation speeds. On the other hand, the recombination of carriers at the surface occurs by the capture of electrons in trap states with lower energy, thereby releasing phonons [46] and increasing the temperature.

## 3 Bowtie cavity side-coupled to a waveguide

**Figure 5:**
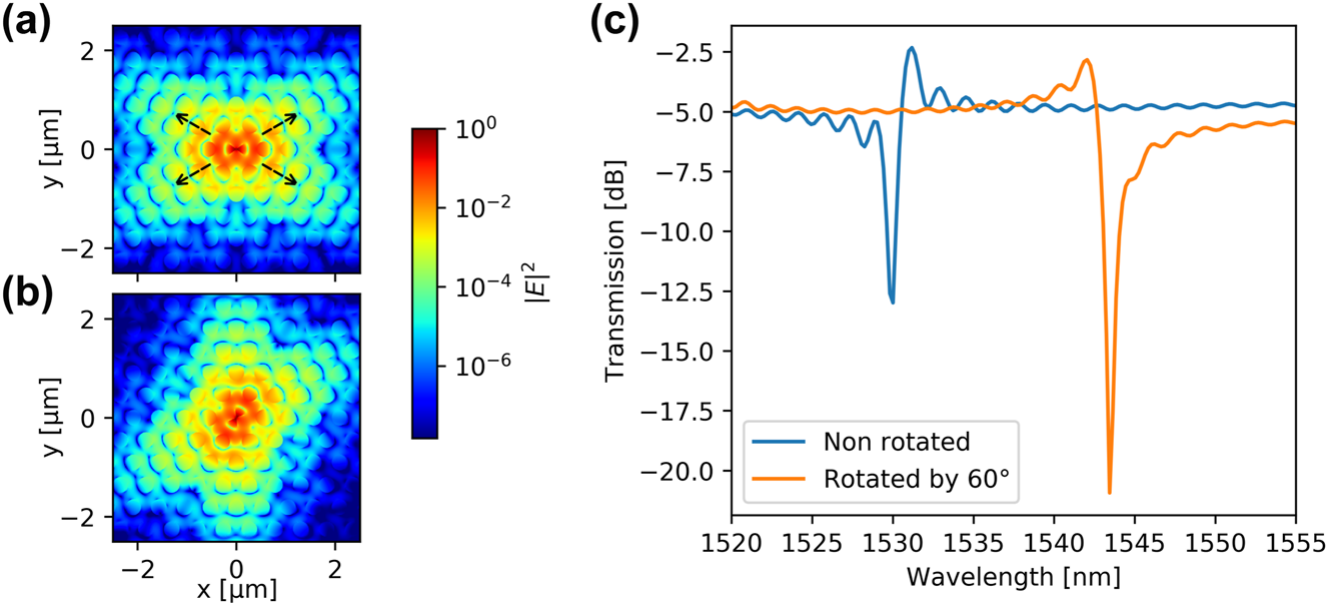
Electric field profile |**E**|^2^ for the bowtie mode in logarithmic scale for (a) non-rotated cavity, and (b) cavity rotated by 60°. The dashed arrows indicate the direction of the lobes of the mode field (c) simulated transmission of a device with rotated and non-rotated cavity.

In this section, the H1 bowtie cavity is side-coupled to a photonic crystal waveguide for coupling light in and out of the structure and simultaneously realize an asymmetric Fano resonance that improves the swicthing contrast. As explained in Ref. [25], the interference between the continuum of modes in the waveguide and the discrete mode of a side-coupled nanocavity leads to a Fano resonance. The frequency-dependent transmission through the structure displays the typical characteristics of a Fano resonance, where, in particular, the asymmetry can be controlled through the incorporation a partially transmitting element in the waveguide [25], [31]. Two changes were made to the cavity-waveguide structure of the H1 bowtie cavity compared to the H0 cavity. The first one is bringing the waveguide closer to the cavity, hence increasing the spatial overlap between the waveguide and the cavity modes. The second one is the angular orientation of the bowtie cavity. The bowtie mode has an evanescent field oriented at 30° angle compared to the *x*-axis of the cavity, as shown in Figure 5(a) by the dashed arrows. Since an efficient interaction between the cavity and the waveguide modes requires a good spatial overlap, a 60° rotation of the bowtie cavity is implemented to improve the coupling [47]. As shown in Figure 5(b), the lower tail of the evanescent field is now vertically oriented, ensuring good overlap with a parallel waveguide. The choice of the rotation angle is fixed by the 60° rotational symmetry of the photonic crystal. We noticed that the coupling between the bowtie mode and the waveguide is very weak for the non-rotated case, leading to very small extinction ratio of the transmission spectrum shown in Figure 5(c).

**Figure 6:**
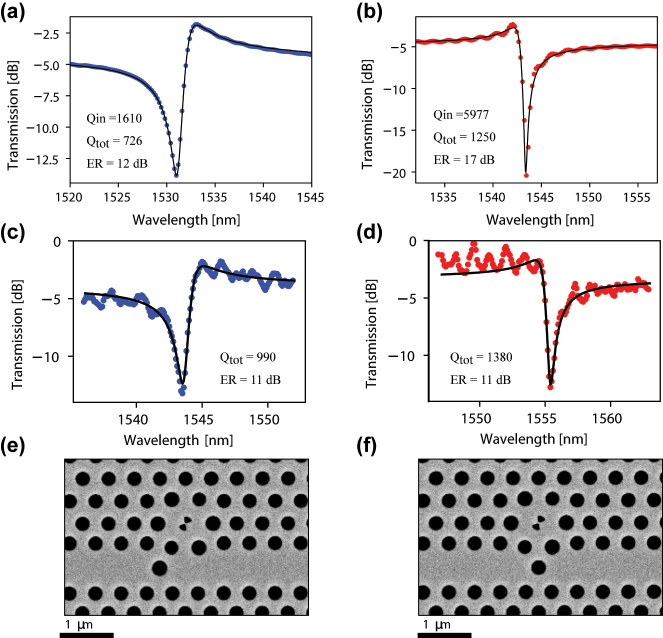
Simulated linear transmission of the H1 bowtie device side-coupled with a waveguide exhibiting (a) blue-detuned and (b) red-detuned asymmetric Fano lineshapes. The blue/red dots show FDTD simulation results, whereas the black solid line shows the prediction of temporal-coupled-mode theory using the fitted parameter values given in the figures. Measured transmission spectrum of (c) blue-detuned, and (d) red-detuned H1 bowtie devices obtained using a Santec-550 tunable laser source. The solid line is the result obtained by fitting to temporal-coupled-mode theory with the indicated parameters. Scanning electron microscope (SEM) images of the fabricated (e) blue-detuned, and (f) red-detuned photonic crystal Fano structures based on bowtie cavities.

The final design is chosen with a rotation of the bowtie by 60°, and a spacing of 2 photonic crystal rows between the center of the bowtie and the center of the waveguide. The SEM pictures shown as Figure 6(e) and (f) illustrate this design choice as well as the specific placement of the PTE in the waveguide leading to blue- or red-detuned resonances. Indeed, by positioning the PTE symmetrically or anti-symmetrically with respect to the vertical axis of the H1 cavity, the phase of the coupling constant between the cavity and the waveguide is changed and lead to a different Fano resonance [48]. The respective lineshapes are simulated using FDTD and shown in Figure 6(a) and (b), for the blue and red-detuned resonances, respectively. Using TCMT, the simulated transmission spectrum is fitted by taking into account the waveguide transmission as well as the wavelength dependent scattering of the PTE. We thus extract total quality-factor values, *Q*_tot_, of 726 and 1,250, and intrinsic quality factors, *Q*_in_, of 1,610 and 5,977 for the blue- and red-detuned resonances, respectively. A drastic drop of the intrinsic quality factor is observed between the value calculated through the field relaxation in the intrinsic cavity shown in Figure 3, and the value extracted from the fitting of the transmission lineshape. This deterioration of *Q*_in_, and the extinction ratio, is due to the addition of the waveguide in close proximity of the cavity, which modifies its resonant properties. In the case of the H0 cavity, the waveguide is typically placed 3 photonic crystal rows from the cavity, leading to a much smaller perturbation. Despite these slightly low extinction ratios of the current designs, we proceed to the fabrication of the devices with H1 bowtie cavities. The fabrication of the devices follows a procedure similar to the one we reported earlier in Ref. [43].

We observe asymmetric Fano lineshapes in the linear transmission measurements as shown in Figure 6(c) and (d). We find that cavities with designed hole radius of 133 nm are spectrally located in the C-band. We observe extinction ratios of ≈11dB for both blue and red detuned resonances. By fitting the measured transmission with TCMT, we obtain *Q*_tot_ of 990 for the blue-detuned mode and 1,380 for the red-detuned mode.

## 4 All-optical switching characterization

**Figure 7:**
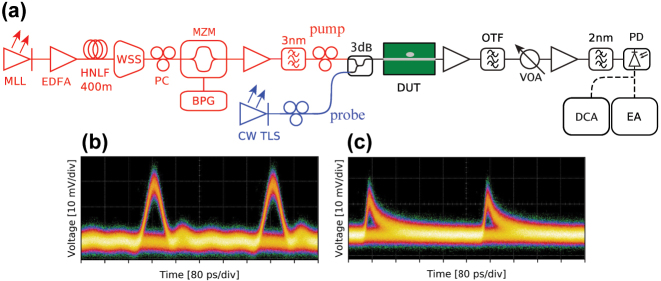
Wavelength conversion. (a) Schematic diagram of the measurement setup used for the wavelength-conversion experiment. The red-colored part of the set-up is used for generating the pump signal, while the blue-colored part is used for generating the probe signal. Eye diagrams measured using (b), a 10 Gbps receiver module, and (c), a 70 GHz bandwidth photodiode. In both cases, the periodicity is 400 ps, corresponding to a bit rate of 2.5 Gbps.

To investigate the dynamical switching properties of the fabricated structures, wavelength conversion experiments are carried out. Figure 7(a) shows the schematic diagram of the experimental setup. Light from a mode-locked laser (MLL), centered around 1,545 nm at 10 GHz repetition rate, is amplified using an erbium-doped fiber amplifier (EDFA) and then launched into a 400 m long highly nonlinear fiber (HNLF) for generating a supercontinuum of light covering the entire C-band. A wavelength-selective switch (WSS) is then used to spectrally carve out the optical spectrum of the pump signal, which is positioned around the peak of the Fano resonance. A pseudo-random bit sequence from a bit-pattern generator (BPG) is used as modulating input signal to a Mach–Zehnder intensity modulator (MZM) to generate a train of on-off keying modulated pump pulses. A continuous wave (CW) tunable laser source (TLS) is used to generate a probe signal spectrally located around the minimum transmission of the Fano resonance. The pump and probe signals are polarization controlled and combined using a 3 − dB coupler before being coupled to the device under test (DUT). Here, the blue-detuned bowtie cavity based Fano resonance of Figure 6(c) is used as the DUT. After the DUT, an optical tunable filter (OTF) is used to filter-out the pump signal. The resulting wavelength-converted signal is detected using a photodiode, and analysed using a digital communication analyser (DCA), and an error analyser (EA). A variable optical attenuator (VOA) is used to control the power of the signal arriving at the photodiode.

The bowtie devices are operated with a low duty-cycle pump pulse at 2.5 Gbps as we observed a large heating of the cavity when operated at 10 Gbps. This is due to the large volume of air regions with low thermal conductance around the cavity, where hot carriers are generated by two-photon absorption. Additionally, the spatial proximity of the optical mode to the etched surfaces of the bowtie increases the probability of surface recombination which also generates heat [46].

By carefully tuning the pump power as well as the wavelength of the probe, we successfully observe the modulation of the CW probe signal. Figure 7(b) and (c) show eyediagrams measured using a 10 Gbps receiver module, and a 70 GHz photodiode, respectively. Although all-optical switching can be observed, we could not quantify the expected carrier relaxation speed enhancement from the eyediagrams. We can, however, observe a fully relaxed cavity after about 240 ps and state that the drop in total quality factor compared to the H0 cavity design, combined with a large reduction of the extinction ratio from >25dB to about 11 dB, reduce the modulation contrast as well as the recovery speed of the modulated signal during all-optical switching operation. Indeed, the average slope of the asymmetric resonance between the minimum and maximum of transmission is smaller at 7.7 dB/nm for the bowtie mode compared to the previously characterized H0 based devices reaching 24.4 dB/nm. Additionally, a strong oxidation of the device is observed when pumped. Due to the strong thermal heating and oxidation of the surface of the semiconductor material, the bowtie devices are susceptible to the amount of optical power coupled to the cavity. It can easily be damaged by a sudden locking of the resonance to the pump due to thermal effects, inducing a spike in the cavity power.

## 5 Conclusions

We designed, fabricated, and experimentally characterized a bowtie nanonocavity which simultaneously features strong spatial light confinement and engineered Fano resonances for improved all-optical switching. The nanocavity is based on a photonic crystal point-defect (H1) cavity containing a rotated bowtie, which realizes a Fano resonance by coupling the discrete mode of the nanocavity to the continuum of modes in a waveguide. We have demonstrated both blue and red-detuned asymmetric Fano lineshapes with extinction ratios around 11 dB. All-optical switching was investigated in a wavelength conversion experiment performed at 2.5 Gbps. While the bowtie nanocavity confines light to a mode volume that is five times smaller than the best H0 or H1 point-defect cavities, we also found that the structure is more susceptible to heating and has reduced extinction ratio compared to H0 nanocavities. The thermal issues can be reduced by surface passivation, e.g. employing a few nanometer thick layer of alumina using atomic layer deposition (ALD) [34]. In addition, better control of the Fano resonances can be achieved by improving the fabrication accuracy as well as implementing thermo-optic tuning elements.

In conclusion, the combination of Fano resonances and strong light localization in subwavelength nanocavities offers new and promising opportunities for realizing all-optical control at ultra-low power levels. Further investigations are needed on the role of surface recombination and the design of structures which are less susceptible to heating. The suggested structures are also of interest for Fano lasers [49], where they can be used to implement nonlinear mirrors, as well as diode structures [7].
